# Exploring the link between serum uric acid and endometriosis: a cross-sectional analysis utilizing NHANES data from 1999-2006

**DOI:** 10.3389/fendo.2025.1536300

**Published:** 2025-04-15

**Authors:** Haiwei Chen, Yuling Chen, Xiaotong Chen, Lixin Tang, Jiaqi Liu, Wen-Jing Shi, Yu-Hua Ou

**Affiliations:** ^1^ Department of Clinical Medicine, The Second Clinical College of Guangzhou Medical University, Guangzhou, China; ^2^ Department of Gynecology, The Second Affiliated Hospital of Guangzhou Medical University, Guangzhou Medical University, Guangzhou, China

**Keywords:** endometriosis, serum uric acid, health care, risk factors, medical research

## Abstract

**Background:**

Substantial impacts on the female reproductive system have been definitively linked to heightened levels of serum uric acid. However, evidence directly linking increased serum uric acid levels to endometriosis in women remains sparse, and the precise characteristics of this influence are still not fully understood.

**Objective:**

To explore the exact relationship between serum uric acid and endometriosis.

**Study design:**

Referencing the data accumulated from the National Health and Nutrition Examination Survey (NHANES), this study covers the period from 1999 to 2006, conducted an analysis of 5,162 female participants aged 20 to 54 years (representing a sample size of approximately 66,927,890 women). The study adopted a cross-sectional methodology to delve into the tie between serum uric acid and the prevalence of endometriosis. Utilizing rigorous methodologies, including weighted multivariable logistic regression models, subgroup analyses, and statistical methodologies for smooth curve fitting.

**Results:**

A positive association was found between continuous serum uric acid and the risk of endometriosis (OR = 1.25, 95% CI [1.09, 1.44], P = 0.003). At the same time, women in the highest quartile had a 133% higher risk of endometriosis compared with women with the lowest quartile of uric acid (OR=2.33,95%CI [1.28, 4.23], P=0.009). At the same time, smooth curve fitting also found a linear positive correlation between serum uric acid and endometriosis. There was no heterogeneity in subgroup analysis.

**Conclusion:**

The study indicates a strong link between increased serum uric acid levels and the appearance of endometriosis in women. Specifically, women with elevated uric acid levels face a higher likelihood of developing endometriosis.

## 1 Introduction

Endometriosis is a typical gynecological condition that affects roughly 10%–15% of women of childbearing age (1). The main symptoms are pain, menstrual abnormalities, and discomfort during sexual intercourse (2). Endometriosis has also been shown to be associated with infertility, affecting up to 50% of all endometriosis patients with infertility (3). In addition, although its malignant rate is relatively low (approximately 0.3%-1.6%), its potential risk of deterioration cannot be ignored (4). Endometriosis seriously affects the wellness of women. The etiopathogenesis of endometriosis has not yet been clarified, which is why its clinical diagnosis and treatment are hindered (5). Previous studies have pointed to a variety of possible factors, including retrograde menstrual blood implantation (6), molecular genetic alterations (7), inflammation (8), immune regulation (9), and hormones (10).

Moreover, one of the cruxes of endometriosis is inflammation. Patients diagnosed with endometriosis demonstrated significantly heightened levels of inflammatory cytokines, specifically interleukin-1 beta (IL-1β), interleukin-6 (IL-6), interleukin-8 (IL-8), vascular endothelial growth factor, CCL2, CCL5, and tumor necrosis factor-alpha (TNF-α). These pro-inflammatory cytokines activate the NF-κB, PI3K, and Akt pathways, exacerbating the inflammatory response (11–13).

The paramount importance of uric acid, which serves as the ultimate product of pyrimidine metabolism in organisms, transcends its sole function as an antioxidant substance. Uric acid holds a fundamental and indispensable role in the scavenging of oxygen radicals and regulation of immunity to promote development, and it also plays an integral role in our bodies (14). However, high concentrations of uric acid have become independent risk factors for many diseases such as nephropathy (15), cardiovascular disease (16), and diabetes (17). Elevated uric acid concentrations result in the formation of sodium urate (MSU) around cells, which in turn activates ASC, recruits human cysteine protease-1, and binds to NLR family pyridine structural domain protein 3 (NLR3) to form NLR3 inflammatory vesicles. This process converts MSU-produced pro-IL-1 to IL-1β, triggering an inflammatory response (18, 19). Accordingly, uric acid serves as a definitive biomarker indicative of inflammatory activities (20, 21).

A considerable volume of scholarly research has unequivocally established a potent connection between inflammation and endometriosis. Regrettably, there has been an absence of scholarly research endeavors to examine the correlation between uric acid levels (22) and endometriosis. The development of online databases has facilitated advances in identifying disease markers (13, 22–26). Hence, the authors embarked on a meticulous and comprehensive analysis of the possible association between uric acid and endometriosis, utilizing a wealth of data sourced from the National Health and Nutrition Examination Survey. This scholarly investigation introduces novel perspectives and substantial contributions to the domain of diagnosing and managing endometriosis, thereby augmenting our understanding of the pertinent diagnostic and therapeutic methodologies.

## 2 Material and methods

### 2.1 Data provenance and examined population

For our cross-sectional analysis, we utilized data extracted from four periods of the National Health and Nutrition Examination Survey (NHANES), which covered the timeframes of 1999-2000, 2001-2002, 2003-2004, and 2005-2006. The aggregate sample size for these periods amounted to 41,474 participants. The NHANES serves as a broad, cross-sectional epidemiological examination, incorporating a typical sample of the whole population. The National Center for Health Statistics (NCHS), which is under the authority of the US Centers for Disease Control and Prevention (CDC), conducts this survey. This study adhered to NHANES laws and guidelines for all analyses. After thoroughly searching and screening the NHANES database, 41,474 participants from 1999 to 2006 were identified. However, only women with an age range of 20–54 years were eventually included. Initially, the NHANES database questioned women aged between 20 and 54 years, inquiring if they had been made aware of their endometriosis diagnosis. In addition, the age bracket most susceptible to endometriosis falls within the childbearing years (typically 20 to 50 years old), where its symptoms (like menstrual pain and impairment of fertility) are most acute. By setting the lower age boundary at 20, we exclude prepubertal and adolescent women who generally have a lower endometriosis incidence and face diagnostic complexities. Conversely, by setting the upper age boundary at 54, we include women in the pre-perimenopausal phase, thereby minimizing the impact of postmenopausal hormonal alterations on our findings. Individuals with incomplete information regarding self-reported endometriosis, serum uric acid levels, and covariates were excluded. The study eventually incorporated a volume of samples equivalent to 5,162 female participants, with 357 of them being women diagnosed with endometriosis, and the remaining 4,805 women being confirmed as not having the condition.

**Figure 1:**
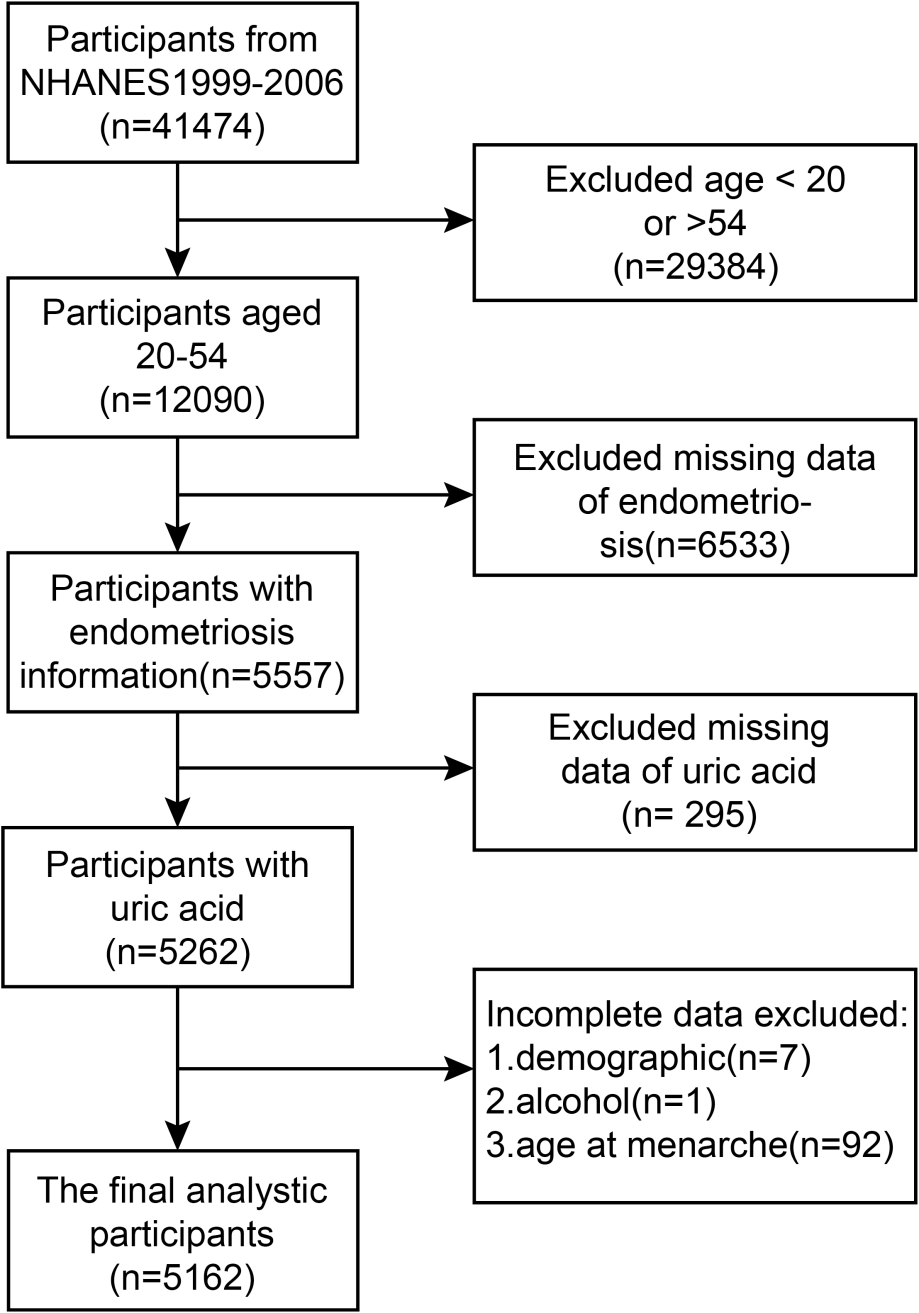
A formal flow diagram illustrating the screening process utilized in the National Health and Nutrition Examination Survey (NHANES) spanning from 1999 to 2006.

And a flowchart is presented in **Figure 1** to elucidate the screening process utilized.

### 2.2 Variables

The exposure variables in this study were serum uric acid and endometriosis. Serum uric acid level, the independent variable, was measured as part of a routine serum biochemical profile using the Beckman Synchron LX20 timed endpoint method. In this process, uric acid oxidase catalyzes uric acid, and then the absorbance is determined colorimetrically to compute the uric acid value. NHANES utilized a standardized method for examining biological samples from 1996 to 2006. This involved using the Beckman Synchron LX20 analyzer for uric acid levels, along with reagents and calibrators that met NIST criteria. Throughout this period, the equipment, techniques, and laboratory conditions remained unchanged. Comprehensive information on quality assurance and control measures can be found on the NHANES website. Before each investigative session, the instruments were carefully calibrated, and stringent quality control assessments were conducted. Thus, the measurement of serum uric acid levels remains reliable. Serum uric acid levels were categorized into four quartiles for statistical analysis: 0.5-3.5 mg/dL for the first quartile (Q1), 3.6-4.2 mg/dL for the second (Q2), 4.3-5.0 mg/dL for the third (Q3), and 5.1-9.6 mg/dL for the fourth (Q4). The reason is that the typical reference range for women’s serum uric acid levels is usually bracketed between 2.4 and 6.0 mg/dL. In this particular study, a classification was adopted that extended from 0.5 to 9.6 mg/dL, including ranges like 0.5-3.5, 3.6-4.2, 4.3-5.0, and 5.1-9.6 mg/dL, which collectively represent the entire spectrum from abnormally low uric acid levels (hypouricemia) to abnormally high levels (hyperuricemia). The quartile divisions (Q1 to Q4) were closely aligned with the 25th, 50th, and 75th percentiles of the NHANES population, providing an accurate depiction of the natural distribution of serum uric acid levels. Participants identified as female by the site-based household surveyors of the NHANES agency were asked questions about reproductive health in the Mobile Examination Center (MEC) exams across four NHANES cycles. Endometriosis was diagnosed through self-reporting based on the “rhq360” questionnaire, which was given at the MEC. The questionnaire asked patients, “Have you ever gotten a diagnosis of endometriosis from a healthcare professional or another authorized medical provider?” If participators answered “yes,” they were classified as a case group with endometriosis and asked about their age at diagnosis; those who responded “no” had no endometriosis.

### 2.3 Other covariates

The continuous covariates encompassed in the analysis are chronological age, body mass index (BMI), which is determined by dividing an individual’s weight measured in kilograms by the square of their height measured in meters, yielding a result expressed in kilograms per square meter (kg/m²). the poverty-to-income ratio (PIR), total serum cholesterol concentration, direct quantification of high-density lipoprotein cholesterol (HDL), concentrations of low-density lipoprotein cholesterol (LDL), creatinine levels, and age at menarche. Categorical covariates encompassed education level, ethnicity/race, marital status, alcohol consumption (defined as consuming at least 12 drinks annually), pregnancy history (whether the individual has ever been pregnant), diabetes history (as diagnosed by a healthcare professional), and smoking history (defined as smoking at least 100 cigarettes in a lifetime). Comprehensive details regarding these covariates can be found on the NHANES website.

### 2.4 Analytical statistics

To compare the characteristics of demographics, reproductive health, smoking and alcohol consumption between patients with endometriosis and non-endometriosis patients, we performed a descriptive analysis of the various included variables. Regarding continuous variables, the data are articulated as mean values appended with their respective standard errors (SE); conversely, for categorical variables, the data are delineated in terms of percentages. Subsequently, to explore the correlation between serum urate concentrations and endometriosis, we employed weighted univariate and multivariate logistic regression analyses as the methodological framework to accomplish the predefined objectives. Taking into account the NHANES database’s elaborate sampling methodology, we adopted weighted univariate analysis to address sampling bias and adjust for discrepancies in sampling probabilities among diverse population subsets. This approach allowed us to decrease sampling error, enhance the reliability and external validity of our estimates, and ultimately formulate study conclusions that better reflect the overall population. In conducting our multivariate logistic regression analysis, we crafted three models based on a binary logistic framework, where endometriosis was designated as the dependent variable. These models integrated serum uric acid and various demographic attributes as independent variables. We subsequently determined the adjusted odds ratio (Adjusted OR) for each variable and evaluated their statistical impact on endometriosis. In the execution of multivariate logistic regression analyses, the following models were meticulously constructed: (1) Model One, which omitted the inclusion of covariate adjustments; (2) Model Two, incorporating nuanced adjustments for the demographic variables of age and race; and (3) Model Three, in which all the covariates were adjusted to ascertain the relationship between endometriosis and serum uric acid. Subgroup analyses adopted multilevel multivariate logistic regression based on the fully adjusted model to identify stratified associations between blood uric acid and endometriosis, along with interaction tests conducted on the subgroups. Additionally, smooth curve fitting was applied to the fully adjusted models to further analyze whether the linkage between serum uric acid amounts and the presence of endometriosis is linear. In our investigation, we adopted a statistical significance threshold of p < 0.05, meaning that results were considered statistically meaningful only when the calculated p-values fell below this predetermined level.

## 3 Results

### 3.1 Baseline features

**Table 1** details the baseline characteristic differences among the study participants. The research study enrolled 5,162 participants in total, all aged between 20 and 54 years, with a specific subset of 357 participants being diagnosed with endometriosis based on medical evaluation. Statistical analysis demonstrated significant disparities between the endometriosis cohort and the non-endometriosis cohort across a range of variables, encompassing age and racial demographics, serum total cholesterol concentration, levels of low-density lipoprotein (LDL), serum creatinine content, educational background, marital status, history of pregnancy, and smoking history (P < 0.05). Specifically, in contrast to the non-endometriosis cohort, patients with endometriosis tended to be older, non-Hispanic white, had higher educational levels, and had higher levels of serum creatinine, LDL, and total cholesterol. They exhibited a higher likelihood of having a history of pregnancy and smoking. When considering serum uric acid as a continuous variable, the median serum uric acid level for patients with endometriosis was (4.81 ± 0.09), compared to (4.46 ± 0.03) for those without endometriosis. The difference in question proved to be statistically notable, as evidenced by a P-value that fell below 0.001. Moreover, upon partitioning the sample into quartiles for analysis, a statistically significant variation in the levels of serum uric acid was observed between the two comparison groups, yielding a P-value of 0.002.

**Table 1:** Weighted participant characteristics according to endometriosis diagnosis assembled from the National Health and Nutrition Examination Survey (NHANES) data points between 1999 and 2006.

Characteristics	Endometriosis (n=357)	Non-Endometriosis (n=4805)	P-value
Age (years), Mean (S.E)	40.22 ± 0.64	36.93 ± 0.31	**<0.001**
Poverty-to-income ratio, Mean (S.E)	3.21 ± 0.16	2.97 ± 0.05	0.161
BMI (kg/cm^2^), Mean (S.E)	28.45 ± 0.47	28.08 ± 0.20	0.463
Total cholesterol (mg/dl), Mean (S.E)	204.86 ± 3.25	194.66 ± 1.10	**0.005**
Direct HDL-cholesterol (mg/dl), Mean (S.E)	56.19 ± 1.60	56.99 ± 0.45	0.634
LDL-cholesterol (mg/dl), Mean (S.E)	122.10 ± 2.74	114.74 ± 1.09	**0.015**
Creatinine (mg/dl), Mean (S.E)	69.28 ± 2.69	62.59 ± 0.31	**0.018**
Age at menarche (years), Mean (S.E)	12.40 ± 0.17	12.66 ± 0.04	0.138
RACE, n (%)			**<0.001**
Mexican American	30 (1.44%)	1216 (8.56%)	
Other Hispanic	7 (1.78%)	249 (5.70%)	
Non-Hispanic White	244 (84.06%)	2144 (67.12%)	
Non-Hispanic Black	62 (8.75%)	982 (12.89%)	
Other Race - Including Multi-Racial	14 (3.96%)	214 (5.74%)	
Education, n (%)			**0.044**
Less than high school	42 (10.57%)	1201 (16.85%)	
High school	315 (89.43%)	3604 (83.15%)	
Marital status, n (%)			**0.021**
Married	224 (69.85%)	2645 (58.67%)	
Divorce	50 (12.06%)	401 (9.93%)	
Separated	17 (3.99%)	212 (3.70%)	
Never married	62 (14.09%)	1409 (27.71%)	
Had at least 12 alcoholic drinks 1 year, n (%)			0.432
yes	244 (71.20%)	2885 (67.40%)	
no	113 (28.80%)	1920 (32.60%)	
Pregnant, n (%)			**0.025**
Yes	307 (87.52%)	4076 (81.55%)	
No	50 (12.48%)	729 (18.45%)	
The doctor told you have diabetes, n (%)			0.678
Yes	13 (3.19%)	216 (3.80%)	
No	344 (96.81%)	4589 (96.20%)	
Smoked at least 100 cigarettes in life, n (%)			**<0.001**
Yes	167 (58.43%)	1796 (40.89%)	
No	190 (41.57%)	3008 (59.11%)	
Uric acid (mg/dl), Mean (S.E)	4.81 ± 0.09	4.46 ± 0.03	**<0.001**
Uric acid (quartile),n (%)			**0.002**
Q1 (0.5-3.5)	61 (10.17%)	1159 (19.95%)	
Q2 (3.6-4.2)	87 (23.21%)	1069 (21.35%)	
Q3 (4.3-5.0)	90 (25.44%)	1327 (29.27%)	
Q4 (5.1-9.6)	119 (41.18%)	1250 (29.42%)	

BMI represents body mass index; direct HDL-cholesterol pertains to direct high-density lipoprotein cholesterol; LDL-cholesterol signifies low-density lipoprotein cholesterol. The abbreviation Mean (S.E) indicates the arithmetic mean and standard error, respectively. Quantities expressed as n (%) pertain to numbers and percentages. Statistically significant values are denoted in the [bold] typeface.

### 3.2 The link between serum uric acid concentrations and the prevalence of endometriosis

**Figure 2:**
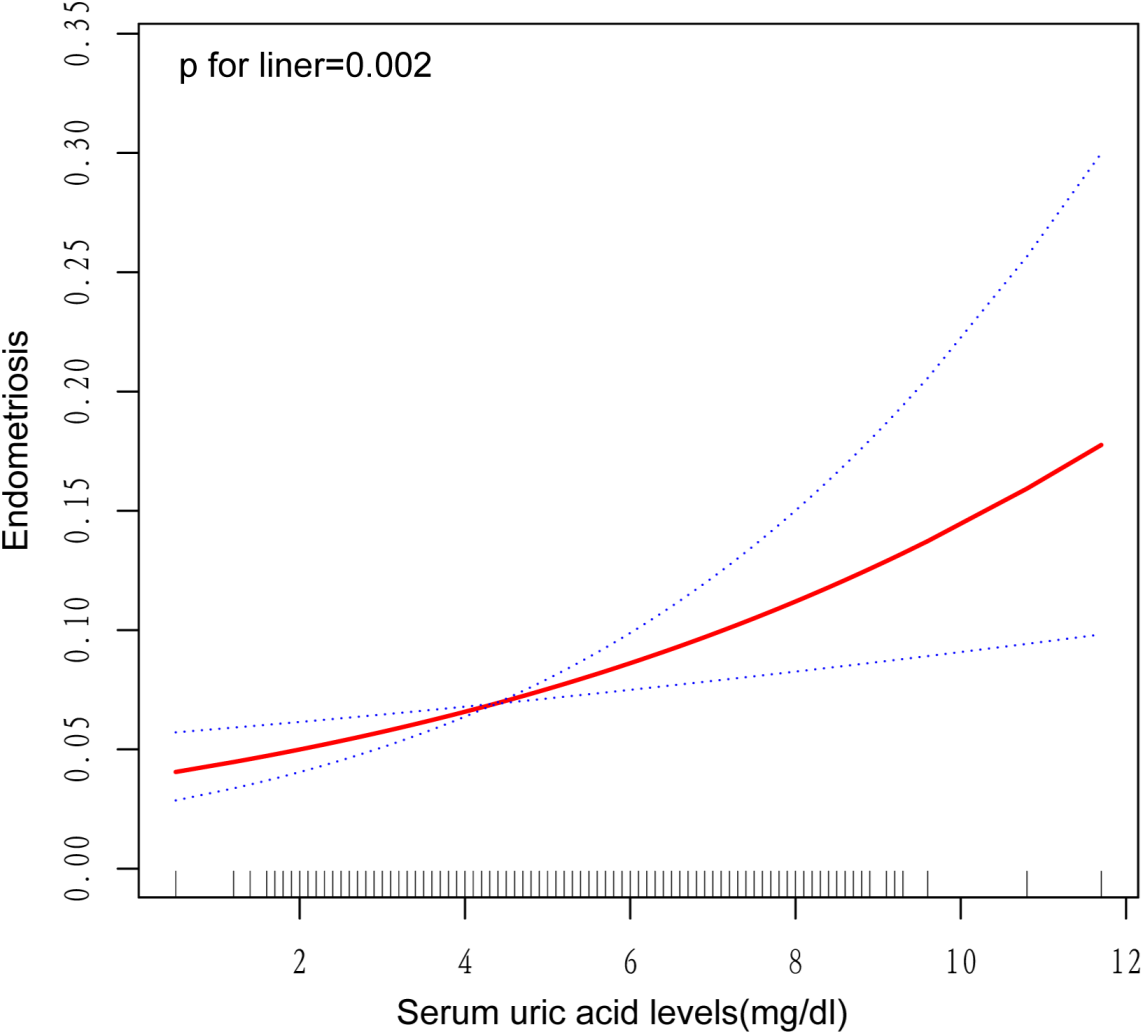
Analysis of endometriosis odds ratio by uric acid levels.

The statistical analysis presented in **Table 2** demonstrates a notable correlation between a rising level of serum uric acid, measured continuously, and an increased susceptibility to endometriosis in women, as demonstrated by notable differences across three analytical models. Specifically, within the parameters of the unadjusted statistical model, the odds ratio (OR) about the prevalence of endometriosis was established at 1.29, with a 95% confidence interval (CI) spanning from 1.12 to 1.50. The statistical significance of this association was confirmed, as indicated by a P-value of 0.001. Within the context of the adjusted model, which incorporated factors such as age and race, the odds ratio (OR) for the incidence of endometriosis elevated to 1.30, with a 95% confidence interval (CI) of 1.13 to 1.48 being reported. The statistical significance of this correlation was established, with a P-value of less than 0.001 serving as confirmation. After incorporating additional confounding variables into the fully adjusted model, the calculated odds ratio (OR) for endometriosis was 1.25, accompanied by a 95% confidence interval (CI) spanning from 1.09 to 1.44. This result suggested a statistically notable link between elevated levels of serum uric acid and the prevalence of endometriosis, as evidenced by a P-value of 0.003. When analyzing serum uric acid as a categorical variable (quartiles), Q2 and Q4 were significantly positively associated with endometriosis when contrasted with Q1 in all models. After fully adjusting for potential confounding factors, women whose serum uric acid levels fell within the highest quartile exhibited a 133% higher probability of developing endometriosis in contrast to individuals in the lowest quartile (OR=2.33, 95% CI: 1.28-4.23, P=0.009). Additionally, a significant trend of increasing endometriosis risk with elevating serum uric acid levels was observed across all three models. Furthermore, the graphical representation in **Figure 2**, featuring a smoothly fitted curve, distinctly demonstrates a positive and straight-line relationship between serum uric acid levels and the prevalence of endometriosis.

**Table 2:** The calculations were performed to determine the unadjusted and adjusted odds ratios (ORs), with weighting applied, for serum uric acid levels, their quartiles, and the associated risk of endometriosis.

	Model 1		Model 2		Model 3	
	OR (95% CI)	P value	OR (95% CI)	P value	OR (95% CI)	P value
Uric acid (mg/dl)	1.29 (1.12, 1.50)	**0.001**	1.30 (1.13, 1.48)	**<0.001**	1.25 (1.09, 1.44)	**0.003**
Uric acid quartile						
Q1 (0.5-3.5)	1.00 (1.00, 1.00)	1.000	1.00 (1.00, 1.00)	1.000	1.00 (1.00, 1.00)	1.000
Q2 (3.6-4.2)	2.13 (1.19, 3.82)	**0.014**	2.14 (1.19, 3.83)	**0.014**	2.15 (1.17, 3.95)	**0.018**
Q3 (4.3-5.0)	1.70 (0.89, 3.27)	0.115	1.63 (0.85, 3.14)	0.148	1.48 (0.73, 2.99)	0.284
Q4 (5.1-9.6)	2.74 (1.56, 4.82)	**<0.001**	2.60 (1.49, 4.53)	**0.001**	2.33 (1.28, 4.23)	**0.009**
P for trend		**0.001**		**0.002**		**0.031**

Statistically significant values are denoted in the [bold] typeface.

Model 1: This model excluded any accommodations for covariates.

Model 2: This model included adjustments for both age and racial factors.

Model 3: Adjustments in this model encompassed age, race, age at menarche, marital status, pregnancy history, smoking status, alcohol consumption, diabetes history, cholesterol levels (total), creatinine concentrations in refrigerated serum samples, LDL-cholesterol levels, HDL-cholesterol levels (measured directly), glycated hemoglobin levels, and body mass index (BMI).

### 3.3 Subgroup analysis exploration

To ascertain more precisely the correlation strength between serum uric acid levels and the prevalence of endometriosis, we embarked on an extensive series of subgroup analyses, taking into consideration a variety of covariates, namely age, educational qualification, marital status, body mass index (BMI), total cholesterol, pregnancy history, and smoking history, as delineated in **Table 3**. The study found no statistically notable interactions between the specified covariates and the levels of serum uric acid, as demonstrated by P-values for interaction that were greater than 0.05. Notably, except for subgroups with ages less than 30 years and BMIs less than 18.5 kg/m², the OR values for all other subgroups were greater than 1, revealing a robust and positive relationship observed between higher levels of serum uric acid concentrations and augmented risk of endometriosis. This result further underscores that the link between serum uric acid and endometriosis remains unaffected by factors such as age, marital status, educational background, body mass index (BMI), total cholesterol levels, pregnancy history, or smoking history.

**Table 3:** Subgroup and interactive analyses of endometriosis and serum uric acid levels from NHANES, weighted.

	OR (95%CI)	P-value	P-interaction
Age(years)			0.612
<30	0.96 (0.55, 1.66)	0.880	
30~40	1.30 (0.98, 1.71)	0.075	
>=40	1.30 (1.08, 1.56)	0.008	
Education			0.937
Less than high school	1.29 (0.91, 1.84)	0.167	
High school	1.27 (1.06, 1.51)	0.013	
Marital status			0.577
Married	1.36 (1.12, 1.64)	0.003	
Divorce	1.07 (0.77, 1.48)	0.682	
Separated	1.13 (0.52, 2.48)	0.763	
Never married	1.05 (0.64, 1.72)	0.855	
BMI(kg/cm^2^)			0.166
<18.5	0.61 (0.17, 2.12)	0.439	
18.5~25	1.01 (0.76, 1.34)	0.960	
>=25	1.60 (1.09, 2.34)	0.022	
Total cholesterol(mg/dl)			0.966
<=200	1.27 (1.02, 1.58)	0.039	
>200	1.28 (1.05, 1.56)	0.022	
Pregnant			0.240
Yes	1.23 (1.04, 1.45)	0.019	
No	1.66 (1.06, 2.59)	0.033	
Smoked at least 100 cigarettes in life			0.492
Yes	1.33 (1.10, 1.60)	0.006	
No	1.16 (0.87, 1.55)	0.329	

Subgroup analyses were undertaken to ascertain the impact of interactions between covariates and serum uric acid concentrations on the prevalence of endometriosis. The significance of these interactions was evaluated using logistic regression analysis, with the resulting p-values serving as indicators of statistical significance.

## 4 Discussion

The current research is a groundbreaking cross-sectional analysis, meticulously designed to investigate the potential link between uric acid levels and the prevalence of endometriosis symptoms. In order to thoroughly and impartially evaluate the relationship between endometriosis and serum uric acid levels, we employed a multifaceted methodology that includes multifactorial logistic regression analysis. Our findings indicate a notable link where higher levels of serum uric acid correspond with a greater prevalence of endometriosis. Remarkably, even after accounting for various influencing variables through adjustment procedures, the correlation remained statistically notable.

Thus far, there have been limited scholarly investigations examining the association between endometriosis and serum uric acid concentrations. However, numerous reports have documented a correlation between disorders affecting the female reproductive system and uric acid levels. Research has demonstrated that blood uric acid levels can be used as a marker to predict complications affecting both the mother and fetus in women who have been diagnosed with preeclampsia (27, 28). Additionally, leukocytes may influence the severity and progression of preeclampsia, potentially through the regulation of uric acid levels (29). The blood uric acid/creatinine proportion is considered to be an additional risk indicator for polycystic ovarian syndrome in obese women (30, 31). Moreover, higher uric acid levels were associated with a greater chance of developing metabolic syndrome among women, regardless of whether they were premenopausal or postmenopausal (32, 33). The published literature offers convincing proof that an elevated blood uric acid level correlates with greater severity in illnesses impacting the female reproductive system. Our research findings are consistent with these outcomes.

The precise mechanisms linking uric acid levels to endometriosis remain insufficiently understood. Our study converted uric acid from a continuous variable to a categorical variable (quartiles). In all three models, compared with Q1 (0.5-3.5 mg/dL), Q2 (3.6-4.2 mg/dL) and Q4 (5.1-9.6 mg/dL) showed a significant positive correlation with endometriosis. The combined smooth curve fitting indicated a linear positive correlation between serum uric acid levels and the risk of endometriosis, suggesting that the risk of endometriosis increases continuously with the elevation of uric acid levels, rather than a risk jump occurring only at a certain critical value (such as the upper limit of the normal range). Therefore, even though Q2 (3.6-4.2 mg/dL) is within the normal range, its positive correlation with Q1 (0.5-3.5 mg/dL) still conforms to the overall linear trend, suggesting that high levels of uric acid within the normal range may not be entirely “safe”. Q4 (5.1-9.6 mg/dL) includes individuals with levels exceeding the normal upper limit (>6.0 mg/dL), and the risk further increases, indicating that excessively high uric acid may exacerbate the risk. However, the risk of Q2 exists independently, suggesting that the effect of uric acid may exist throughout the range from low to high, rather than only appearing within the abnormal range. The risk of Q3 (4.3-5.0 mg/dL) is not significant, which may be due to the presence of local nonlinear effects, confounding factors, or data noise. Despite contradictions, the increased risk in the Q2 and Q4 ranges still has important clinical significance. The normal range of serum uric acid for clinical females (2.4-6.0 mg/dL) is based on population distribution or biochemical metabolic health definitions and may not directly reflect the pathological threshold for specific diseases.

This study indicates that for endometriosis, the effect of uric acid may be continuous and dose-dependent, and even if it does not exceed the normal range, higher levels may still be associated with disease risk. This is linked to elevated uric acid promoting oxidative stress, inflammatory responses, and vascular formation, and is associated with changes in sex hormone levels. A study conducted by Elena and colleagues demonstrated that asymptomatic hyperuricemia is linked with elevated levels of oxidative stress and a rise in inflammatory mediators in non-pregnant adult women (18, 34). Studies have shown a direct connection between elevated levels of uric acid and the inflammatory response, revealing that these heightened levels assist in developing inflammatory vesicles, aiding in the conversion of pro-IL-1 to IL-1β (35). In turn, miR-302a is upregulated (36). The expression of MiR-302a inhibited the upstream promoter of nuclear receptor chicken ovalbumin transcription factor II (COUP-TFII), thereby releasing the inhibition of COX-2 gene and promoting the increase of COX-2 and its downstream product prostaglandin (PGE2) (37). Through several routes, this process aggravates the inflammatory response (38, 39), which in turn encourages changes in the uterine milieu and necrotic apoptosis, which eventually contributes to the pathophysiology of endometriosis (40). Additionally, IL-1β is recognized for its role in amplifying the inflammatory response by upregulating MMP12, MMP1, PAI2, and other cytokines and growth factors, thereby fostering neovascularization as well as matrix remodeling, which are implicated in the development of endometriosis (41–43). Additionally, research indicates that serum uric acid enhances the migration of human vascular smooth muscle cells (HVSMC), while simultaneously inhibiting the migration of human umbilical vein endothelial cells (HUVEC). This suggests that serum uric acid might facilitate vascularization in endometriotic tissue (7). In a recent study, it was demonstrated that uric acid levels exhibit an inverse relationship with the hormones estrogen and progesterone. It is important to note that progesterone enhances the renal excretion of uric acid. Therefore, individuals with endometriosis and elevated uric acid levels may demonstrate a correlation with increased estrogen levels and reduced progesterone levels in their bodies. The reduction in progesterone levels leads to decreased renal uric acid clearance, which is reflected in elevated serum uric acid levels (44, 45). Additionally, another study suggested that uric acid may lower total testosterone levels in women (46, 47). Since testosterone can exert anti-inflammatory effects by promoting energy storage, a reduction in testosterone may impair the body’s ability to counteract the pro-inflammatory effects of estrogen, potentially exacerbating the inflammatory response to ectopic endometrial tissue (48–50). Our findings align with prior research.

Therefore, our findings suggest that uric acid levels can be included in a comprehensive risk assessment for people with endometriosis symptoms or at high risk (such as family history, and infertility patients). Even if uric acid is in the normal range, individuals near the upper limit (such as Q2 and above) may need closer monitoring or early intervention. At the same time, it is recommended that patients with endometriosis maintain a low normal level of uric acid (close to Q1) through lifestyle adjustments (such as a low-purine diet, weight control, and moderate exercise), especially for women with existing symptoms such as dysmenorrhea and chronic pelvic pain. At the same time, because the current evidence is only observational, it has not been proven that lowering uric acid can directly prevent or treat endometriosis. In the future, it is necessary to further explore the mechanism of uric acid in endometriosis (such as oxidative stress, and inflammatory activation) and determine whether it is a pathogenic factor or a concomitant phenomenon. The causal relationship between uric acid level and disease risk can be verified through a series of intervention experiments and clinical studies, and the risk threshold of different subgroups can be refined.

In our results, we found that patients with endometriosis were more likely to have a history of smoking and pregnancy and were more likely to be married than those without endometriosis. Even if these variables do not affect the relationship between serum uric acid and endometriosis, the significant effects of smoking, pregnancy, and marital status on endometriosis are still noteworthy. Regarding the relationship between pregnancy and endometriosis, Pregnancy is considered to have a positive effect on the relief of endometriosis and its associated painful symptoms, not only because the suspension of ovulation reduces bleeding from ectopic endometrial tissue, but also because of the various metabolic, and hormonal regulation, immune response, and angiogenesis changes that accompany pregnancy (51). Studies have found that an increase in the number of pregnancies reduces the risk of endometriosis (52). Nevertheless, studies have also shown that pregnancy in women with endometriosis does not necessarily lead to relief of symptoms or a reduction in the size of endometriosis lesions. In some cases, the risk of malignant transformation of ovarian endometriosis lesions may even be observed. The occurrence of these complications may be related to chronic inflammatory processes, tissue adhesion, progesterone resistance, and abnormal gene expression involved in embryo implantation (53). At the same time, spontaneous abdominal blood accumulation during pregnancy is also thought to be associated with the risk of endometriosis (54).In addition, studies have found that ectopic pregnancy is associated with an increased risk of endometriosis (55). This is consistent with our findings. However, at present, the specific association between pregnancy and endometriosis and its mechanism still needs further research. In addition, our study found that married women have an increased risk of endometriosis in their marital status, and we believe that this may play a mediating role in pregnancy after marriage as discussed earlier. The specific mechanism needs further study.

The specific mechanism by which smoking can increase the risk of endometriosis is not fully understood, but tobacco smoke contains a complex mixture of chemicals, including many reactive oxygen species and nitrogen substances (ROS and RNS), and the possible reason is that smoking promotes the overproduction of oxygen free radicals (56), The balance between ROS production and antioxidant defense is disrupted, leading to oxidative stress in patients with endometriosis (57), Oxidative stress is thought to be a factor in local damage to the peritoneal mesothelium, which provides a site for the attachment of ectopic endometrial cells and promotes the apoptotic process of these cells (58–60). This is consistent with our findings. However, it is interesting to note that many previous studies have shown that smoking can reduce the risk of endometriosis, often because endometriosis is an estrogen-dependent disease, and tobacco changes the metabolism of estradiol, leading to increased production of inactive catechol estrogens, which have anti-estrogenic effects (61, 62). Therefore, the specific association between smoking and endometriosis still needs further investigation.

This investigation presents several notable advantages. Our study is a trailblazing effort. It is the inaugural utilization of the NHANES database to conduct a thorough exploration of the possible link between endometriosis and serum uric acid levels. NHANES utilizes a comprehensive, multi-stage probability sampling methodology to conduct annual surveys and evaluations encompassing roughly 5,000 individuals, reflecting the demographics of the entire nation. In this research, we analyzed data from 5,162 women, weighted to represent a population of 66,927,890 women. Employing a multivariate regression model, we delved into subgroup analyses to examine the varying connections between serum uric acid levels and endometriosis among different populations, segmented by age, BMI, pregnancy status, and racial classifications. The findings of these analyses provide evidence that supports a positive relationship between endometriosis and serum uric acid levels.

However, it bears mentioning that the research is not without its limitations. Notably, the cross-sectional design employed means that serum uric acid levels and endometriosis are assessed concurrently at the time of the study. The simultaneity of the survey complicates the assessment of the temporal relationship between potential causes and effects. Secondly, For our study, we utilized the NHANES database focusing on women aged 20-54 with endometriosis, excluding those outside this age range. While this exclusion might have led to the neglect of a tiny fraction of early or late-diagnosed endometriosis cases, representing a minimal proportion, it still introduced a selection bias. Therefore, the conclusions drawn from this study are exclusively applicable to women of reproductive age and necessitate further validation in future studies involving adolescents and elderly individuals. Additionally, due to the high rate of endometriosis loss associated with the covariate age, there may be a degree of bias in the results. Lastly, we cannot completely rule out the possibility of sampling errors in NHANES data. In light of these limitations, future research needs to further validate our findings through large-scale prospective cohort studies.

## 5 Conclusion

The study indicates a strong link between increased serum uric acid levels and the appearance of endometriosis in women. Specifically, women with elevated uric acid levels face a higher likelihood of developing endometriosis. The insights gained from this research can serve as a reference for preventing disorders of the female reproductive system, including endometriosis. Although uric acid management is not currently included in the guidelines for the prevention and control of endometriosis, based on the public health pathway, uric acid testing can be added to the routine physical examination of women of reproductive age (especially those with dysmenorrhea, infertility, or metabolic syndrome) to optimize screening for high-risk groups. Nevertheless, additional fundamental research is required to substantiate our findings and elucidate the underlying physiological mechanisms involved. We should focus on the molecular network of uric acid regulation, identify its potential as a target for prevention and treatment, and further study the influence of uric acid on the pathophysiology and analysis of various stages of endometriosis.

## Data Availability

The original contributions presented in the study are included in the article/supplementary material. Further inquiries can be directed to the corresponding authors.
